# Baseline value of intrahepatic HBV DNA over cccDNA predicts patient’s response to interferon therapy

**DOI:** 10.1038/s41598-017-05242-y

**Published:** 2017-07-19

**Authors:** Di Mu, Fang-Chao Yuan, Yu Chen, Xiao-Yan Jiang, Liang Yan, Ling-Yu Jiang, Jian-Ping Gong, Da-Zhi Zhang, Hong Ren, Yong Liao

**Affiliations:** 1 Key Laboratory of Molecular Biology for Infectious Diseases, Ministry of Education, Chongqing, P.R. China; 2 Institute for Viral Hepatitis, Chongqing Medical University, Chongqing, P.R. China; 3 Department of Infectious Diseases, The Second Affiliated Hospital, Chongqing Medical University, Chongqing, P.R. China; 4 Department of Hepatobiliary Surgery, The Second Affliated Hospital, Chongqing Medical University, Chongqing, P.R. China

## Abstract

Methodology for accurate quantification of intra-hepatic cccDNA has long been a technical challenge, yet it is highly desired in the clinic. Here, we developed a sensitive method for quantification of intrahepatic cccDNA in liver biopsies from patients, which allowed to predict patient’s response to interferon therapy at baseline. Twenty-five patients with HBeAg+ CHB were recruited and liver biopsies were obtained at baseline and 1-year after interferon treatment, respectively. Both intrahepatic cccDNA and HBV DNA were absolutely quantified by a droplet digital PCR amplification system. Patients were categorized as either responder or non-responder group based on their HBeAg status 1-year after interferon therapy. Levels of both intrahepatic HBV DNA and HBV cccDNA were significantly reduced after interferon treatment among the responders, but not the non-responders, in comparison with their levels at baseline. Baseline values of intrahepatic HBV DNA over cccDNA significantly correlated with patient’s response to PEG-IFN therapy (P = 0.000). In addition, HBeAg seroconversion also correlates with a significant reduction in intrahepatic pgRNA production among the responders after interferon therapy (P = 0.030). In conclusion, our results suggest that baseline value of intrahepatic HBV DNA over cccDNA may be a preferable indicator for selecting appropriate patients for IFN-based therapy in the clinic.

## Introduction

Hepatitis B virus (HBV) infection remains a major global health problem. Over 350 million people are chronically infected with HBV^1^. Chronic HBV infection (CHB) increases the risk of developing liver cirrhosis and hepatocellular carcinoma, and causes 0.5~1.2 million deaths annually^2, 3^. Currently, two types of anti-viral therapies are commonly available in clinical practice for patients with CHB infection: nucleos(t)ide analogues (NA) and pegylated interferon-α (PEG-IFN)^4^. Although NA-based therapy is effective and convenient, these agents cannot eliminate the intracellular HBV replication template termed cccDNA or covalently closed circular HBV DNA. Alternatively, PEG-IFN is a first-line treatment for management of certain patients with CHB. Due to the severe side effects, only a fraction of patients are eligible for interferon treatment, and only <10% of patients responded persistently to interferon-α treatment and achieved clinical cure^1, 5^.

Currently, there is no reliable biomarker available for predicting patient’s response to IFN therapy. Theoretically, level of intra-hepatic cccDNA is the most direct prognostic indicator of response to anti-viral therapy. Earlier studies demonstrate that cccDNA quantification from liver biopsies of infected patients is a useful marker for assessment of treatment efficacy^4, 6–8^. An expanded application of this useful marker is hampered by the technical challenge in measurements of cccDNA from the tiny amount of tissues obtained from liver biopsies^9, 10^. Currently, quantitative PCR (qPCR) is the principal method for monitoring intrahepatic HBV cccDNA^10, 11^. However, this technique needs more than 10^2^ copies of cccDNA to generate a detectable signal, a requirement rarely achieved with biopsy samples, particularly after anti-viral therapy. Therefore, methodology for accurate quantification of intrahepatic cccDNA during anti-viral therapy of CHB patients has long been a challenge.

In our previous report, we introduced a droplet digital PCR (ddPCR)-based amplification system, which provides a sensitive and an accurate quantification of copy numbers of HBV plasmid DNA and the intrahepatic cccDNA in a stable cell line with replicative HBV expression^12^. Here, we applied this methodology to detect intra-hepatic HBV DNA and cccDNA in liver biopsies obtained from CHB patients. The results demonstrate that baseline transcriptional activities of intra-hepatic HBV cccDNA correlate with their differential responses to PEG-IFN treatment among HBeAg (+) CHB patients and may be a useful biomarker for selecting appropriate patients for IFN-based therapy in future clinical practice.

## Results

### Baseline characteristics and categories of patients to PEG-IFN therapy

In this study, a total of 25 patients with HBeAg positive CHB were previously recruited in a randomized trial of peg-interferon (PEGASYS^TM^) at baseline, but only 17 patients were remained one-year after IFN therapy and therefore included in the current analysis [including 12 males and 5 females (age range, 18–50 years)] (Supplementary Fig. S1A). The remaining 8 patients were excluded as patients failed to present to the clinic during follow up and thus their liver biopsies after PEG-IFN therapy were not available for current analysis. The serologic conversion of HBeAg is considered a significant end point for IFN therapy in HBeAg-positive CHB patients and was defined by negative HBeAg and positive HBeAb as described before. Thus, the patients were divided into two groups after 48 week treatment with PEG-IFN: non-responder or HBeAg-positive group (n = 11), and responder or HBeAg-seroconverted group (n = 6). The characteristics of the 17 patients at baseline and 48 weeks after treatment are shown in Table 1.

**Table 1:** Difference of Hepatitis B patients serum parameters between responders and non-responders at baseline or one-year after PEG-IFN therapy.

Characteristics	Non-responders		P	Responders		P
	Baseline	One-year after IFN		Baseline	One-year after IFN	
Age (years) (Mean ± SD)	26.55 ± 4.76			24.50 ± 5.21		
Gender (Male/Female)	9/2			3/3		
Therapeutic	Peg-IFN-alpha (n = 11)			Peg-IFN-alpha (n = 6)		
Drug dosage	180 ug/week			180 ug/week		
Treatment time (weeks)	44.7 ± 5.6			42 ± 6.57		
HBV DNA (Log_10_ IU/mL)	8.03 ± 0.36	5.52 ± 2.15	0.018*	8.28 ± 0.46	2.55 ± 1.42	0.000*
HBsAg (Log_10_ IU/mL)	4.16 ± 1.11	3.61 ± 1.02	0.267	4.45 ± 0.43	1.66 ± 1.18	0.002*
HBeAb (COL)	4.92 ± 2.48	2.72 ± 2.32	0.240	4.55 ± 1.28	0.46 ± 0.53	0.001*
ALT (IU/L)	119.18 ± 60.43	63.73 ± 28.22	0.032*	147.50 ± 99.64	47.17 ± 33.86	0.005*
AST (IU/L)	69.00 ± 38.91	44.36 ± 23.87	0.439	105.83 ± 70.51	38.17 ± 17.93	0.046*
ALB (g/L)	13.30 ± 4.78	9.84 ± 3.07	0.121	12.35 ± 4.79	11.78 ± 4.21	0.240
TBIL (mg/dL)	43.86 ± 2.76	45.44 ± 2.14	0.062	43.58 ± 2.61	45.18 ± 0.90	0.816

*Represent statistical difference.

There were no significant differences between the non-responders and responders with regard to ALT, AST, albumin and total bilirubin, neither at baseline nor after treatment (Table 1). In consistent with previous report^7^, levels of serum HBsAg declined significantly in the responders after 12-week’s PEG-IFN treatment (P = 0.002, Table 1 and Supplementary Fig. S1B,C), but this parameter showed no significant difference in the non-responders after PEG-IFN treatment (P = 0.267, Table 1). Serum ALT decreased significantly after treatment in patients from both groups, however, ALT recovered to normal levels in the responders only. The level of serum AST in patients from responders was higher than that in the non-responders at baseline, however, AST in the responders reduced significantly and returned to normal level after PEG-IFN treatment (P = 0.046, Table 1). Changes in ALB and TBIL were not statistically significant, neither before nor after treatment between the two groups (Table 1).

### A ddPCR-based approach is more sensitive than that of conventional qPCR in detecting intrahepatic HBV cccDNA in liver biopsy

**Figure 1:**
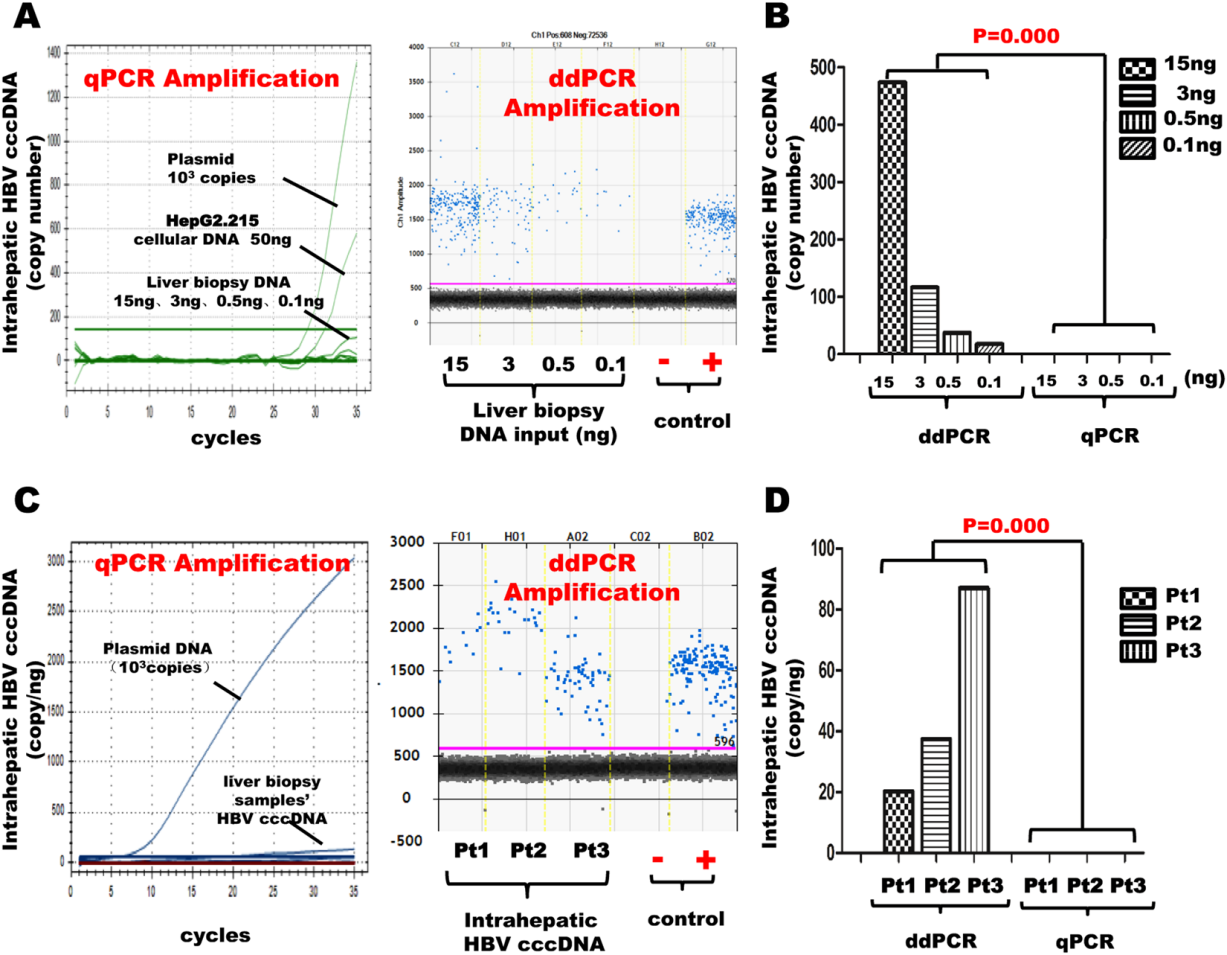
Comparison of cccDNA quantification assay using a ddPCR versus a qPCR amplification system. (**A**) HBV cccDNA amplification with either the qPCR or ddPCR system. (**B**) Quantification of copy numbers of HBV cccDNA detected by either the ddPCR or qPCR system. (**C**) Detection and quantification (**D**) of HBV cccDNA from three different CHB patients by the ddPCR or qPCR system.

To test whether the ddPCR based-methodology is more sensitive than regular qPCR method in detection of HBV cccDNA from liver biopsy specimens, we serially diluted intrahepatic DNA extracted from liver biopsy specimens into 15 ng/ul, 3 ng/ul, 0.5 ng/ul, 0.1 ng/ul, which were then digested with or without PSAD, and amplified, respectively, by either ddPCR or qPCR system. The results showed that HBV cccDNA could be detected by ddPCR with as little as 0.1 ng of the intra-hepatic DNA input, while qPCR failed to detect any HBV cccDNA with as much as 15ng of intra-hepatic DNA input (i.e., ~150 fold difference, Fig. 1A,B). To further evaluate the sensitivity of ddPCR versus that of qPCR in the detection of intrahepatic HBV cccDNA, we measured the levels of intra-hepatic HBV cccDNA in 1ng of the intra-hepatic total DNA (as the template input) from three liver biopsies obtained from CHB patients with serum HBV DNA higher than 10^7^ copies/ml before any anti-viral treatment. The results showed that intra-hepatic HBV cccDNA could be detected in all three biopsy samples by the ddPCR system (Fig. 1C), while qPCR failed to generate a detectable signal in the same set of DNA samples (Fig. 1D). Together, these results demonstrate that ddPCR detects intrahepatic HBV cccDNA from liver biopsy specimens with improved accuracy and sensitivity than that of the qPCR system.

### Treatment with PEG-IFN significantly reduced levels of both intra-hepatic HBV DNA and HBV cccDNA in the responders, but not in the non-responders

**Figure 2:**
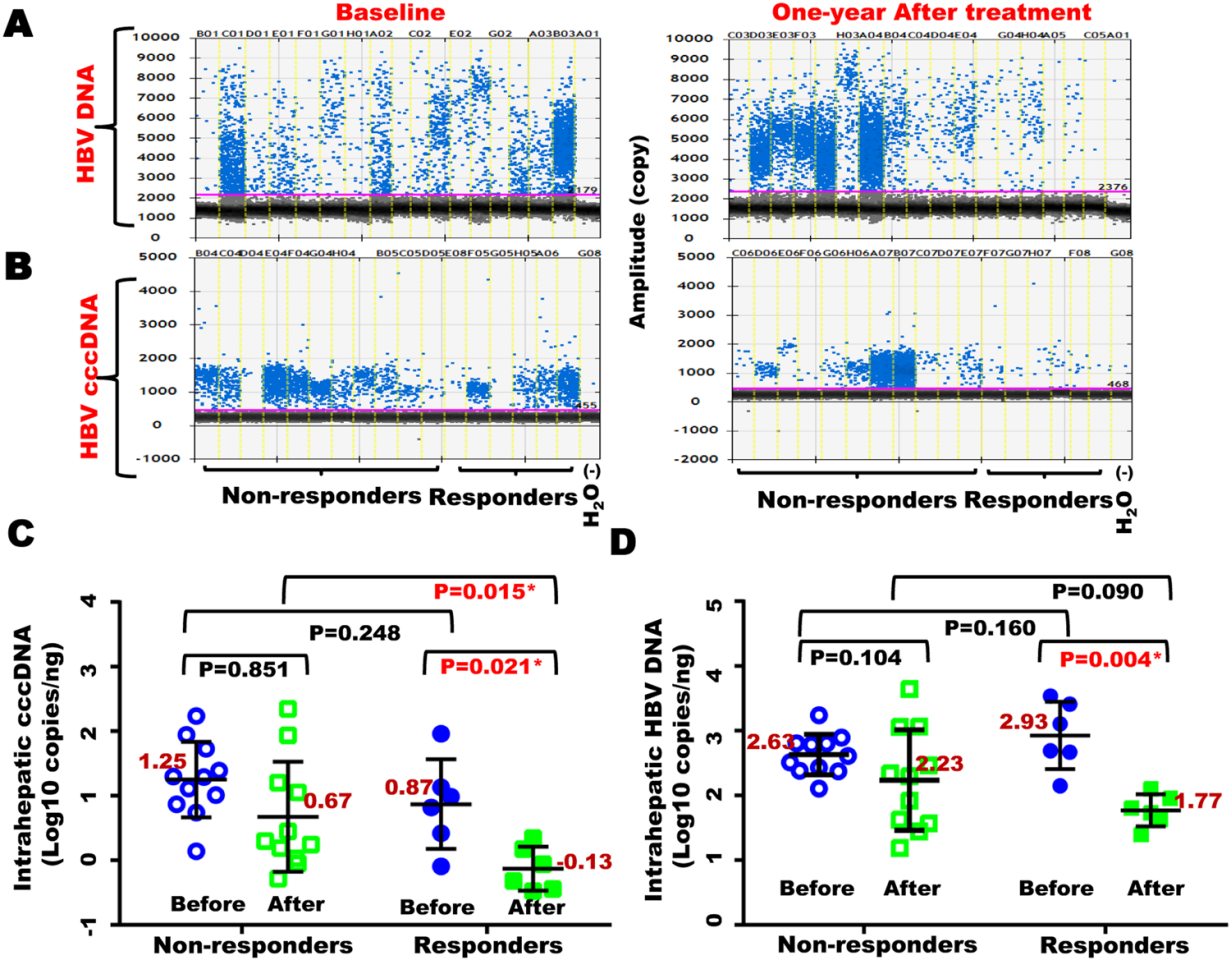
Amplification of HBV cccDNA and HBV DNA by ddPCR in paired liver biopsy specimens from 17 CHB patients at baseline and one-year after treatment with PEG-IFN. (**A**) Amplification of HBV DNA and cccDNA (**B**) by ddPCR system from liver biopsies obtained from CHB patients before and after treatment with PEG-IFN. (**C**) Quantification of intrahepatic HBV cccDNA and HBV DNA (**D**) before and after IFN treatment in responders and non-responders. Before, before treatment; After, after treatment.

Next, we investigated the alterations of intra-hepatic HBV DNA and HBV cccDNA in paired liver biopsies obtained from 17 patients at baseline and one-year after PEG-IFN treatment (Fig. 2A,B). As shown in Fig. 2B, intrahepatic HBV cccDNA were detectable by the ddPCR system in all biopsy specimens obtained at baseline and 1-yr after PEG-IFN treatment respectively, including those biopsy specimens obtained from patients with HBeAg seroconversion. In the non-responder group, mean values of intrahepatic cccDNA and HBV DNA, as expressed as a log_10_ conversion of raw data, were 1.25 ± 0.58 and 2.63 ± 0.31 copies/ng, respectively, at baseline; and these values were reduced to 0.67 ± 0.85 (P = 0.851) and 2.23 ± 0.78 (P = 0.104) (copies/ng), respectively, after 1-year PEG-IFN therapy (Fig. 2C,D, Supplementary Table S1). Compared with mean values of intrahepatic cccDNA and HBV DNA at baseline, both values were significantly decreased one-year after PEG-IFN treatment in the responders, but not the non-responders, and the reductions in the mean value of HBV cccDNA and HBV DNA at baseline and after PEG-IFN treatment within the responders are statistically significant (Fig. 2C,D, Supplementary Table S1; P = 0.021 and P = 0.004, respectively). In comparison with the mean values of HBV cccDNA between responders versus non-responders, there is a significant difference in the levels of intrahepatic HBV cccDNA after PEG-IFN treatment (P = 0.015), but not at baseline (P = 0.851) (Fig. 2C). However, there is no significant difference in the levels of intrahepatic HBV DNA between these two groups, neither at baseline (P = 0.160) nor at 1-year after treatment with PEG-IFN (P = 0.090, Fig. 2D), indicating that the HBeAg seroconversion might associated with PEG-IFN-mediated reduction in HBV cccDNA, instead of HBV DNA itself.

### The values of intrahepatic HBV DNA over cccDNA at baseline correlates with patient’s response to PEG-IFN therapy

**Figure 3:**
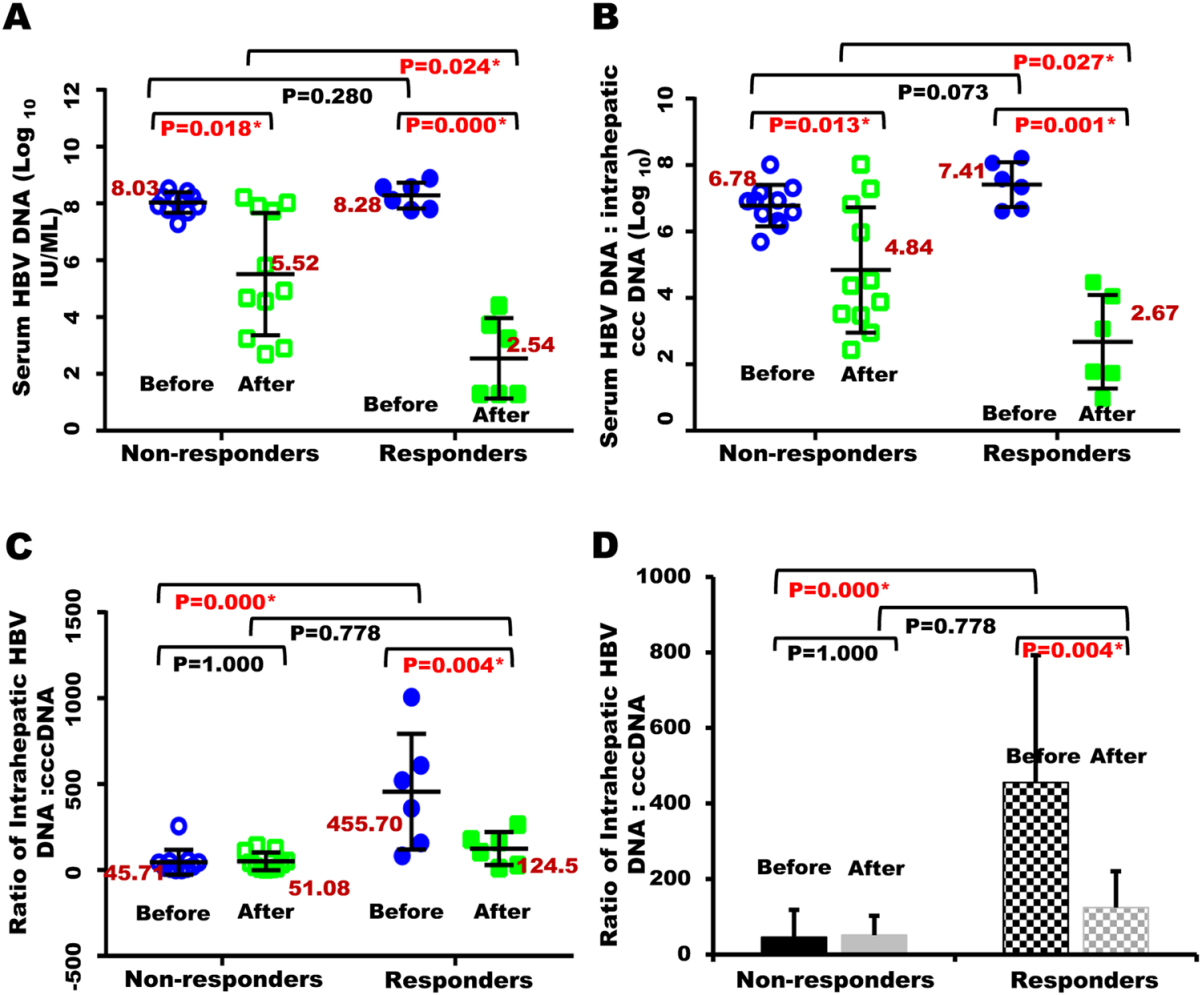
Correlations between levels of intrahepatic (or serum) HBV DNA and cccDNA. (**A**) Relative levels of serum HBV DNA in each individual CHB patient at baseline and after IFN treatment in responders versus non-responders. (**B**) Values of serum [or intrahepatic (**C**)] HBV DNA over intrahepatic HBV cccDNA in each individual CHB patient at baseline and after IFN treatment. (**D**) Mean values of intrahepatic HBV DNA over cccDNA in non-responders versus responders at baseline and after IFN treatment.

Since there is no significant difference in the levels of HBV DNA or cccDNA between these two groups at baseline, we then analyzed the relationship between the levels of intra-hepatic (or serum) HBV DNA and intrahepatic HBV cccDNA at baseline or after PEG-IFN therapy in these patients. Again, no significant difference in the levels of serum HBV DNA was observed between the non-responders and responders at baseline (P = 0.280) (Fig. 3A, Table 1 and Supplementary Fig. S1D). Levels of serum HBV DNA in both groups declined significantly after IFN treatment, but responders had achieved 5–6 logs reduction, which is statistically significant (P = 0.024, Fig. 3A), when compared with the non-responders (around 2~3 logs reduction).

Since the ratio of the intrahepatic HBV DNA: cccDNA may reflect the replicative or transcriptional activity of the intrahepatic cccDNA in HBV virion production in the patients, i.e., how many copies of HBV DNA or viral particles may be reproduced by a cccDNA in an individual patient, we therefore compared the relative ratios of HBV DNA (serum or intrahepatic) over HBV cccDNA in both groups. Again, we noticed that there was no significant difference in the baseline value of serum HBV DNA:cccDNA between the responders and non-responders (P = 0.073, Fig. 3B). However, there was significant difference of reduction in the value of serum HBV DNA:cccDNA after PEG-IFN treatment in either the non-responders or responders (P = 0.013 or P = 0.001, Fig. 3B) in comparison with their baseline values. In addition, values of serum HBV DNA:cccDNA in the responders had a remarkable decrease than that of the non-responders after PEG-IFN treatment (p = 0.027, Fig. 3B).

As mentioned above, levels of both intra-hepatic HBV DNA and cccDNA themselves are comparable between the two groups, yet levels of HBV DNA are relatively higher while levels of cccDNA are lower in the responders than that of non-responders at baseline (Fig. 2D). Indeed, our analysis demonstrate that there is a statistically significant difference in the baseline values of intrahepatic HBV DNA:cccDNA between these two groups (P = 0.000, Fig. 3C,D). Interestingly, there was no significant alteration in the value of HBV DNA:cccDNA within non-responders group between baseline and after IFN treatment (P = 1.000, Fig. 3C,D). There was also no significant difference in the value of HBV DNA:cccDNA between the responders and non-responders after IFN treatment (P = 0.778, Fig. 3C,D). However, compared with the baseline values of intrahepatic HBV DNA:cccDNA, there was a significant reduction in the responders after PEG-IFN treatment (P = 0.004, Fig. 3C,D).

Taken together, the above results suggest that, in comparison with non-responders, the PEG-IFN mediated HBeAg sero-conversion correlated with reduction in the levels of intra-hepatic (or serum) HBV DNA and cccDNA. However, only the baseline values of intrahepatic HBV DNA:cccDNA could potentially predict patient’s response to PEG-IFN therapy, and the higher the better response.

### IFN-induced HBeAg conversion correlates with reduction in intrahepatic pgRNA production

**Figure 4:**
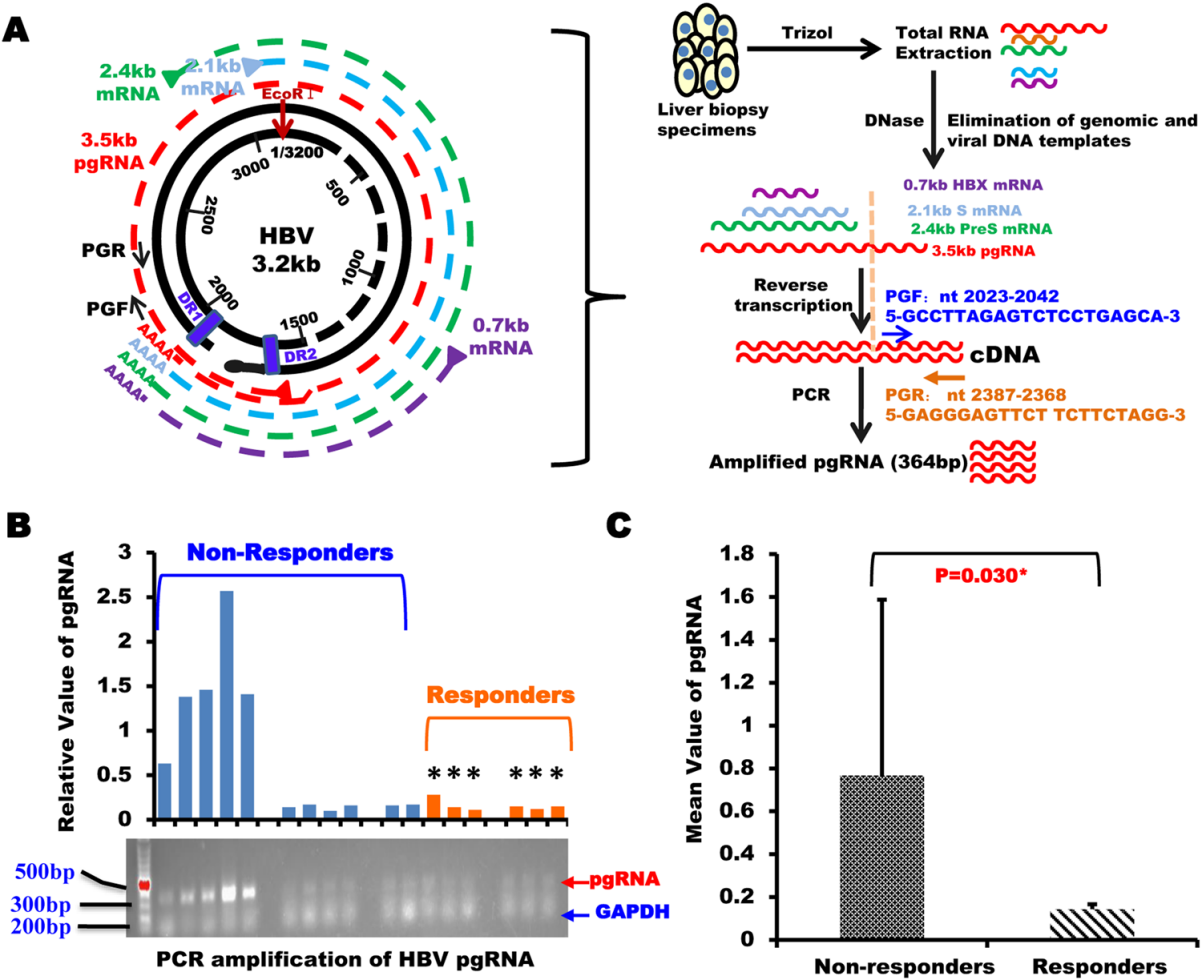
Difference of intrahepatic pgRNA in non-responders versus responders after IFN treatment. (**A**) Flowchart of HBV pgRNA specific amplification procedures. PGF: pgRNA forward primer; PGR: pgRNA reverse primer. (**B**) Agarose gel electrophoresis (lower panel) and quantification (upper panel) of HBV pgRNA products amplified by PCR in liver biopsy after IFN treatment. Note: Patients with HBeAg sero-conversion are marked with “*”. (**C**) Mean values of intrahepatic pgRNA in non-responders versus responders after IFN treatment.

To determine if PEG-IFN-mediated HBeAg sero-conversion is associated with the effect of IFN on repression of intra-hepatic pgRNA transcription from the intrahepatic HBV cccDNA, we extracted total RNA and measured the levels of intra-hepatic HBV pgRNA in the fresh biopsy samples from patients after IFN treatment by the established method (Fig. 4A,B)^10^. As expected, the mean value of intrahepatic HBV pgRNA in the responders was far lower than in the non-responders (P = 0.030, Fig. 4C). We then evaluated the correlations between pgRNA and the intrahepatic HBV DNA and cccDNA. As expected, a close correlation between the levels of intrahepatic HBV DNA and pgRNA was observed in the liver biopsy specimens of all the 17 patients after IFN treatment (R = 0.739, P = 0.001, Supplementary Fig. S2A). When we analyzed the data separately, i.e., within the responders and the non-responders, the non-responders showed a strong correlation between the levels of intrahepatic HBV DNA and pgRNA (R = 0.713, P = 0.014, Supplementary Fig. S2B), however, no such correlation was found within the responders (R = −0.452, P = 0.369, Supplementary Fig. S2B).

Next, we analyzed the potential correlations between HBV pgRNA with intrahepatic HBV cccDNA after PEG-IFN therapy. We observed a statistically significant correlation between intrahepatic HBV pgRNA with HBV cccDNA, when data from the 17 patients were analyzed altogether (R = 0.907, P = 0.000, Supplementary Fig. S2C). Again, when we analyzed the data separately within the responders and the non-responders alone, we could only observe a strong correlation in the dataset obtained from the non-responders (R = 0.921, Pc = 0.000, Supplementary Fig. S2D), but not in the responders (R = −0.148; P = 0.779, Supplementary Fig. S2D). These results imply that PEG-IFN-mediated HBeAg serological conversion may be associated with the direct effect of IFN on the life cycle of cccDNA, cccDNA-based pgRNA transcription, as well as HBV DNA replication.

## Discussion

Incomplete clearance of Intrahepatic HBV cccDNA is the major obstacle for the current anti-viral therapy and also the cause of relapse after sustained anti-viral therapy^4, 13^. Earlier studies demonstrate that cccDNA quantification from liver biopsies of infected patients is a useful marker for assessment of treatment efficacy^7, 8^. In recent years, numerous qPCR-based quantitative methods for detection of HBV cccDNA have been developed, yet a non-qPCR-based rolling circle amplification (RCA) is more widely used and the sensitivity of RCA in detection of HBV cccDNA is much higher than the qPCR-based approaches^14, 15^. However, RCA assay itself is qualitative, not quantitative. More recently, a report from Yuan’s lab demonstrates a highly sensitive and specific ISH assay to visualize intrahepatic HBV DNA and cccDNA at the single-cell level in paraffin-embedded tissue slides from CHB patients^16^. Unfortunately, none of those aforementioned methodologies was able to accurately quantify HBV cccDNA. In the current report, with the application of the ddPCR-based amplification system, we demonstrate an exceptionally higher sensitivity and accuracy in detection of intrahepatic HBV cccDNA. With this novel technology, intrahepatic HBV cccDNA is ~150 fold more sensitive than that of the qPCR-based methods. Thus, this methodology may represent an ideal monitoring assay for HBV cccDNA fluctuation during an anti-viral therapy.

In the clinic, HBeAg sero-conversion is predictive of sustained virologic response to antiviral treatment and is also an ideal endpoint of IFN therapy in HBeAg positive CHB patients^17^. However, HBeAg sero-conversion only occurs in about 30% of CHB patients receiving PEG-IFN therapy. Given the severe toxicity of interferon for most patients and the uncertainty of efficacy in certain patients, a good biomarker for pre-selection of patients for interferon-based anti-viral therapies is urgently needed. By far, numerous surrogate serum biomarkers have been applied to evaluate patient’s response to interferon, such as HBsAg^18, 19^, serum HBV DNA^20^, or hepatitis B core-related antigen (HBcrAg)^21^; however, none of these surrogate markers is able to predict the potential response when patients receiving the interferon therapy. Data presented in the current report suggest that baseline value of intrahepatic HBV DNA over HBV cccDNA, i.e., the transcriptional activity of cccDNA, in liver biopsy specimens may be a promising parameter for selecting patients for interferon-based anti-viral therapy (Fig. 3C). Particularly, patients with a highly and transcriptionally active intrahepatic cccDNA (i.e., higher ratios of HBV DNA:cccDNA) appears more susceptible toward interferon-mediated cccDNA degradation than those with transcriptionally inactive cccDNA (i.e., lower ratios of HBV DNA:cccDNA). Furthermore, the factors that affect the treatment response to PEG-IFN (such as Age, Gender, Intrahepatic HBV DNA and cccDNA, Serum HBV DNA, Intrahepatic HBV DNA/cccDNA, HBsAg, HBeAb, ALT, AST, ALB and TBIL) have been analyzed by multivariate analysis using logistic analysis. Unfortunately, there is no statistical significance among all the factors mentioned above between non-responders and responders groups at baseline, which may be caused by a small sample size (Supplementary Table S2 and Supplementary Table S3).

In conclusion, the ddPCR-based amplification system for the detection of HBV cccDNA may represent a technological breakthrough for accurate quantification and sensitive detection of intrahepatic HBV cccDNA in liver biopsy specimens obtained from CHB patients. This ddPCR-based methodology also enables monitoring fluctuations of all forms of HBV DNA with high sensitivity and accuracy during an antiviral therapy. Although a limited number of patients were enrolled in the current study, we expect that validation with a larger cohort of patients may help to define a cut-off baseline value of intrahepatic HBV DNA:cccDNA for selecting appropriate patients for interferon-based therapy in the clinic.

## Patients and Methods

### Patient Characteristics and Study Design

Twenty-five treatment naïve chronic hepatitis B patients were previously recruited in a randomized trial of peg-interferon (PEGASYS; Roche Pharmaceuticals, Shanghai, China) between May 2013 and January 2015. All patients were enrolled and followed up by the Department of Infectious Diseases, at the Second Affiliated Hospital of Chongqing Medical University, Chongqing, China. Eight patients were either declined for the 2^nd^ biopsy after IFN therapy or lost contact during the follow-up, and the remaining seventeen patients (12 men and 5 women) were included in the current report. The patients were between 18 and 50 years of age and seropositive for HBsAg and HBeAg at baseline. Levels of serum HBV DNA of the enrolled patients are all above 10^7^ copies/mL at baseline. All patients were included in the study documented HBcAg-IgG positive for >6 month before starting treatment. And other viral co-infections are excluded (such as HDV, HCV, HEV and HIV). PEG-IFN was administered at a dose of 180 ug/week for 48 weeks. Paired liver biopsies were collected and snap frozen, at baseline and 48 weeks after treatment, respectively, and stored in liquid nitrogen until analysis. The characteristics of the 17 patients at baseline and 48 weeks after treatment are shown in Table 1.

### Laboratory Measurements

HBsAg/HBsAb and HBeAg/HBeAb were detected by the electrochemiluminescence method with the COBAS e601 automatic electrochemical luminescence immunity analyzer (Roche Diagnostics, Branchburg, NJ). HBeAb and HBeAg values were reported as a signal-to-cut off ratio index: an index of HBeAg below 1.0 indicates a negative reaction and an index of HBeAb below 1.0 indicates a positive reaction. By definition, an HBeAg serologic conversion indicates HBeAg is negative while HBeAb is positive.

Serum HBV-DNA levels were determined by Roche real-time fluorescent quantitative polymerase chain reaction (PCR) (Lightcycler; Hoffman-La Roche, Swiss). The detection limit of this HBV-DNA assay was 20 IU/mL or 116.4 copies/mL.

Total DNA and RNA were extracted from about 10 mg of liver biopsy tissues with TRIzol reagent (Invitrogen, Carlsbad, USA) according to the manufacture protocol. Droplet digital PCR (ddPCR) and real-time fluorescence quantification (qPCR) were applied to quantify HBV DNA and HBV cccDNA as described previously^12^. The QuantaSoft analysis software (Bio-Rad, USA) was used to analyze the ddPCR data, and the concentration of positive droplets were determined by calculating the ratio of the positive droplets over the total droplets combined with Poisson distribution. The qPCR reactions were performed according to the manufacturer’s instructions using CFX96 Touch Real-Time PCR Detection System (Bio-Rad, USA), and the reaction conditions were the same as that of the ddPCR system.

Levels of HBV pgRNA in CHB patients after PEG-IFN therapy were measured by the method as described previously^22^. Briefly, RNA samples were digested using RQ1 RNase-Free DNase (Promega, USA) for 30 min at 37 °C. DNase-treated RNA samples (2ug for each reaction) were reversely transcribed into cDNA with 5 * X All-In-One RT MasterMix (Applied Biological Materials, Canada) and primers (see Fig. 4A). The cDNA were then amplified with the C1000 Thermal Cycler (Bio-Rad, USA) and quantity of the PCR amplified cDNA products was measured by agarose gel electrophoresis and analyzed by the Image Lab software (Bio-Rad, USA).

### Ethical considerations

The study was in accordance with the principles of the 1975 Declaration of Helsinki and was approved by the Ethics Committee of Internal Review Board, 2nd Affiliated Hospital, Chongqing Medical University, Chongqing, P.R. of China. All enrolled patients consented individually and informed consent was obtained from all subjects before acquirement of their liver tissue specimen by needle biopsy.

### Statistical analysis

Statistical analyses were performed with SPSS18.0 software. Serum HBV DNA, biopsy HBV DNA and biopsy HBV cccDNA were subjected to logarithmic conversion for analysis. Data obtained at baseline and 48 weeks after treatment were compared by Paired-sample t-test. Correlation analysis was performed (Pearson Correlation Analysis) between intrahepatic HBV DNA and HBV cccDNA; HBV cccDNA and pgRNA; HBV DNA and pgRNA, as well as serum HBsAg and intrahepatic cccDNA. The difference between data derived from the qPCR amplification versus that from the ddPCR was analyzed by t-test. The factors that affect the treatment response to PEG-IFN were analyzed by multivariate logistic Regression analysis. A two-tailed p value of less than 0.05 was considered statistically significant.

## Electronic supplementary material

Supplementary information
