# Supplementary material for: Baseline value of intrahepatic HBV DNA over cccDNA predicts patient’s response to interferon therapy
Source: Sci Rep. 2017 Jul 19;7:5937. doi: 10.1038/s41598-017-05242-y (PMC5517439; doi:10.1038/s41598-017-05242-y)
Supplement: Supplementary file 1 — Supplementary information [file 41598_2017_5242_MOESM1_ESM.pdf]

Baseline value of intrahepatic HBV DNA over cccDNA predicts patient's response to interferon therapy

Authors: Di Mu[1,2,3]; Fang-Chao Yuan[4]; Yu Chen[1,2]; Xiao-Yan Jiang[1,2]; Liang Yan[1,2,3]; Ling-Yu Jiang[1,2]; Jian-Ping Gong[4]; Da-Zhi Zhang[1,2,3]; Hong Ren[1,2,3]*; Yong Liao[1,2,3]*

Affiliation: [1]Key Laboratory of Molecular Biology for Infectious Diseases，Ministry of Education, PR China

[2]Institute for Viral Hepatitis, Chongqing Medical University, Chongqing, PR China

[3]Department of Infectious Diseases, The Second Affiliated Hospital, Chongqing Medical University, Chongqing, PR China.

[4]Department of Hepatobiliary Surgery, The Second Affliated Hospital, Chongqing Medical University, Chongqing, PR China

***Correspondence:** Yong Liao, Key Laboratory of Molecular Biology for Infectious Diseases (Ministry of Education), Institute for Viral Hepatitis, Department of Infectious Diseases, The Second Affiliated Hospital, Chongqing Medical University, Chongqing, PR China. Phone/Fax：86-23-6389-3780; E-mail: y8982000@yahoo.com; yongliao@hospital.cqmu.edu.cn. Or to: Hong Ren, Key Laboratory of Molecular Biology for Infectious Diseases (Ministry of Education), Institute for Viral Hepatitis, Department of Infectious Diseases, The Second Affiliated Hospital, Chongqing Medical University, Chongqing, PR China. Phone: 86-23-6369-3029, Fax: 86-23-6370-3790; E-mail: renhong0531@vip.sina.com.

Supplementary Table S1. Mean values of HBV DNA and HBV cccDNA in liver biopsy specimens of hepatitis B patients at baseline and one-year PEG-IFN therapy

Characteristics	Non- responders (n=11)	Responders (n=6)	P
**Baseline**			
HBV DNA ($\log_{10}$ copies/ng)	2.63±0.31	2.93±0.52	0.160
HBV cccDNA ($\log_{10}$ copies/ng)	1.25±0.58	0.87±0.63	0.248
HBV DNA: cccDNA	45.71±72.44 (95%CI：-2.96~94.38)	455.65±337.06 (95%CI：101.93~809.38)	**0.000***
**One-year after IFN**			
HBV DNA ($\log_{10}$ copies/ng)	2.23±0.78	1.77±0.25	0.090
HBV cccDNA ($\log_{10}$ copies/ng)	0.67±0.85	-0.13±0.3	**0.015***
HBV DNA: cccDNA	51.08±51.51	124.48±96.01	0.778

*** represent statistical difference，“CI” represent Confidence intervals.**

Supplementary Table S2. Multivariate logistic regression analysis of influence factors associated with different responses at baseline.

Characteristics	Non-responders VS Responders (at baseline)		
	P	HR	95%IC
Age (years)	0.999	0.008	0.000-
Gender (Male/Female)	0.999	0.000	0.000-
Intrahepatic HBV DNA（log_{10} copies/ng）	1.000	2.273E20	0.000-
Intrahepatic cccDNA（log_{10} copies/ng）	0.999	0.000	0.000-
Intrahepatic HBV DNA/cccDNA	0.999	1.092	0.000-2.26E113
Serum HBV DNA(Log_{10} IU/mL)	0.999	0.000	0.000-
HBsAg(Log_{10} IU/mL)	0.999	2.810E8	0.000-
HBeAb (COL)	0.999	0.003	0.000-
ALT(IU/L)*	---	---	---
AST (IU/L)	0.999	5.206E28	0.000-
ALB (g/L)*	---	---	---
TBIL (mg/dL)	1.000	0.717	0.000-

*** represent the variable ALB and ALT are constant for the selected cases and are removed from the analysis by SPSS software.**

Supplementary Table S3. Multivariate logistic regression analysis of influence factors associated with different responses after one year treatment

Characteristics	Non-responders VS Responders (after treatment)		
	P	HR	95%IC
Age (years)	0.999	0.000	0.000-
Gender (Male/Female)	1.000	1237972	0.000-
Intrahepatic HBV DNA（log_{10} copies/ng）	0.999	1.489E35	0.000-
Intrahepatic cccDNA（log_{10} copies/ng）	0.999	0.000	0.000-
Intrahepatic HBV DNA/cccDNA	0.999	1.771	0.000-
Serum HBV DNA(Log_{10} IU/mL)	1.000	0.002	0.000-
HBsAg (Log_{10} IU/mL)	1.000	620.923	0.000-
HBeAb (COL)	0.998	0.000	0.000-
ALT(IU/L)	1.000	0.000	0.000-
AST (IU/L)	1.000	3.768E13	0.000-
ALB (g/L)*	---	---	---
TBIL (mg/dL)*	---	---	---

*** represent the variable ALB and TBIL are constant for the selected cases and are removed from the analysis by SPSS software.**

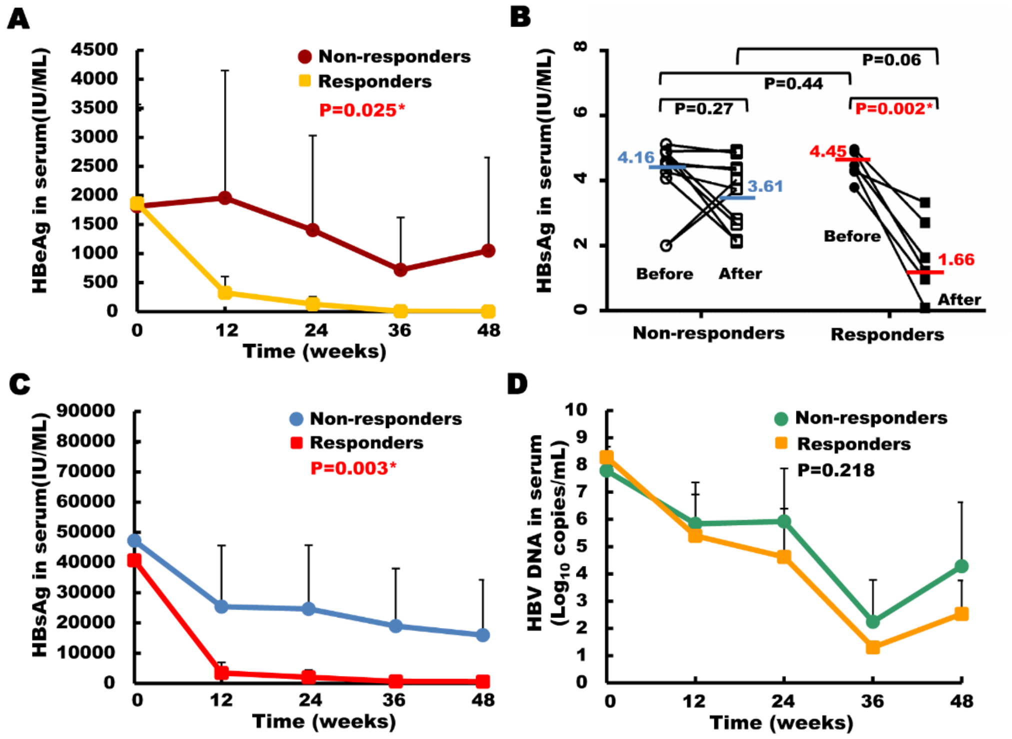

Supplementary Fig.S1. Changes in the level of serum HbeAg, HBsAg and serum HBV DNA during 48-week IFN treatment in responders and non-responders.

(**A**) Changes in the level of serum HBeAg during 48-week IFN treatment in responders and non-responders.

(**B**) The $\log_{10}$ transformed value of serum HBsAg level before and after treatment in responders and non-responders.

(**C**) Changes in the level of serum HBsAg and serum HBV DNA (**D**) during 48-week IFN treatment in responders and non-responders.

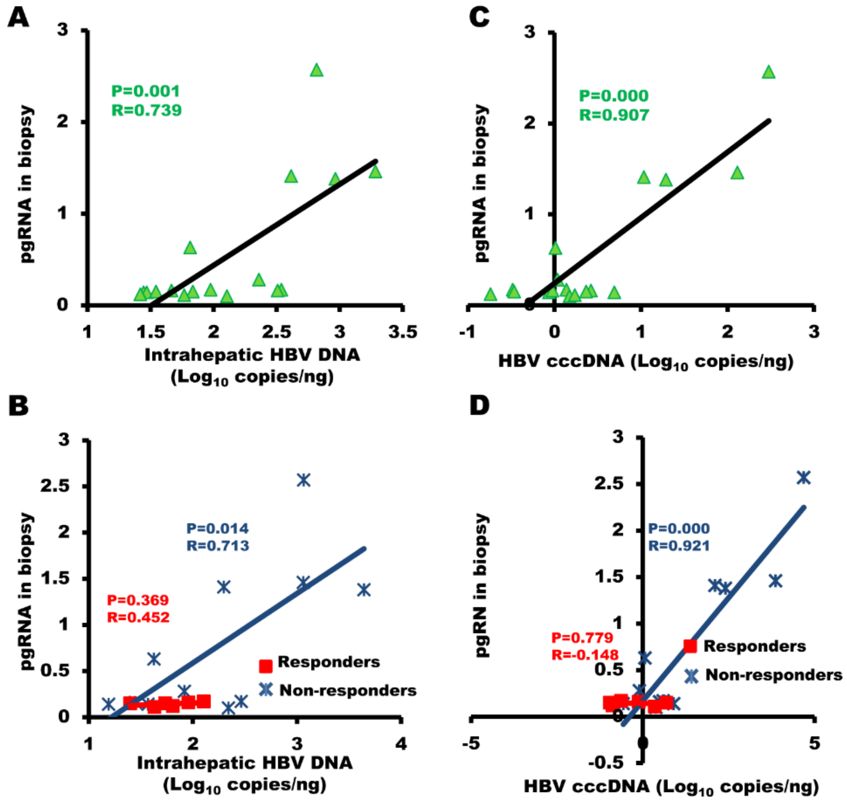

Supplementary Fig.S2. Correlations between intrahepatic HBV pgRNA with HBV DNA and HBV cccDNA

(**A**) Relationship between intrahepatic HBV pgRNA and HBV DNA in all 17 CHB patients after treatment with PEG-IFN. R: coefficient of correlation. P: A two-tailed p value of less than 0.05 was considered statistically significant.

(**B**) Relationship between intrahepatic HBV pgRNA and HBV DNA within responders (■) or non-responders✱).

(**C**) Relationship between intrahepatic HBV pgRNA and HBV cccDNA in all 17 CHB patients after treatment with PEG-IFN.

(**D**) Relationship between intrahepatic HBV pgRNA and HBV cccDNA within responders (■) or non-responders✱).
